# YKL-40 as a Novel Factor Associated with Inflammation and Catabolic Mechanisms in Osteoarthritic Joints

**DOI:** 10.1155/2014/215140

**Published:** 2014-07-15

**Authors:** Tuija Väänänen, Anna Koskinen, Erja-Leena Paukkeri, Mari Hämäläinen, Teemu Moilanen, Eeva Moilanen, Katriina Vuolteenaho

**Affiliations:** ^1^The Immunopharmacology Research Group, University of Tampere School of Medicine and Tampere University Hospital, 33014 Tampere, Finland; ^2^Coxa Hospital for Joint Replacement, P.O. Box 652, 33101 Tampere, Finland

## Abstract

YKL-40 is associated with tissue injury and inflammation, and consequently to diseases in which these mechanisms lead to tissue degradation, for example, asthma and rheumatoid arthritis. The purpose of the present study was to investigate if YKL-40 is also a significant factor in osteoarthritis (OA) by assessing associations of YKL-40 with mediators related to the pathogenesis of OA: cartilage destructing matrix metalloproteinases (MMPs) and proinflammatory cytokines interleukin-6 (IL-6) and interleukin-17 (IL-17). Cartilage, synovial fluid (SF), and plasma samples were obtained from 100 OA patients undergoing total knee replacement surgery. SF levels of YKL-40 (1027.9 ± 78.3 ng/mL) were considerably higher than plasma levels (67.2 ± 4.5 ng/mL) and correlated with YKL-40 released from cartilage samples obtained from the same patients (*r* = 0.37, *P* = 0.010), indicating that YKL-40 is produced by OA cartilage. Interestingly, YKL-40 concentrations in OA SF correlated positively with MMP-1 (*r* = 0.36, *P* = 0.014), MMP-3 (*r* = 0.46, *P* = 0.001), IL-6 (*r* = 0.57, *P* < 0.001), and IL-17 (*r* = 0.52, *P* = 0.010) levels. Moreover, IL-6 and IL-17 enhanced YKL-40 production in human primary chondrocyte cultures. The present study introduces YKL-40 as a cartilage-derived factor associated with mediators of inflammation and cartilage destruction involved in the pathogenesis of OA.

## 1. Introduction

YKL-40, also known as BRP-39, Chi3-l1, and HC-gp39, is a 40 kDa chitinase-like protein without chitinase activity [1–3]. YKL-40 was discovered in 1992 as a product of MG63 human osteosarcoma cell line [4] and cloned and characterized in 1993 as a major secretory product of articular chondrocytes and synovial fibroblasts from patients with rheumatoid arthritis (RA) [5]. After these initial reports, associations with asthma, COPD, liver fibrosis, and cancer have indicated a role for YKL-40 in inflammation and tissue remodeling, but the exact biological activities are yet to be identified [1].

Within joints, YKL-40 is not only produced by articular chondrocytes [2, 6, 7], but also by synovial membrane fibroblast and macrophages [2, 6], as well as by synovial fluid (SF) neutrophils [2]. Further, osteoblasts and primary osteocytes in osteophytes have been shown to express YKL-40 [7, 8]. Volck et al. reported that in osteoarthritic cartilage, YKL-40 is produced in particularly in areas burdened by high biomechanical load, while in normal cartilage none or very sparse positive YKL-40 staining could be detected.

Circulating YKL-40 levels have been shown to be higher in OA patients compared to healthy controls [2, 9, 10]. Also associations to CRP and matrix metalloproteinase (MMP)-3 levels have suggested connections to inflammation and to the pathogenesis of OA [2, 10, 11]. However, the role of YKL-40 in OA joints remains mainly unknown.

OA is a whole joint disease in which proinflammatory and cartilage destructive mediators from joint tissues are secreted into the SF affecting the cartilage [12]. Cartilage degradation, the main feature of OA, is mainly mediated by extracellular matrix degrading MMP enzymes [13]. We hypothesized that YKL-40 is found in OA joints and that it is involved in the pathogenesis of cartilage destruction. In the present study, we aimed to address the hypothesis by measuring the simultaneous levels of YKL-40 in plasma and in synovial fluid as well as to investigate whether YKL-40 in SF is associated with inflammatory and catabolic factors MMP-1, MMP-3, IL-6, and IL-17 in patients with osteoarthritis.

## 2. Materials and Methods

### 2.1. Patients and Samples

The patients in the present study fulfilled the American College of Rheumatology classification for OA [14]. Blood and SF samples and cartilage tissue were obtained from 100 OA patients undergoing total knee replacement surgery as previously described by Koskinen et al. [15]. Plasma and SF samples were stored at −80°C until analyzed. Cartilage samples were processed as described below. The study was approved by the Ethics Committee of Tampere University Hospital, Tampere, Finland, and was conducted in accordance with the Declaration of Helsinki. All patients provided their written informed consent.

### 2.2. Cartilage Cultures

Cartilage cultures were performed as previously described by Koskinen et al. [16]. Briefly, leftover pieces of OA cartilage from knee joint replacement surgery were used. Full-thickness pieces of articular cartilage from femoral condyles and tibial plateaus showing macroscopic features of early OA were removed aseptically from subchondral bone with a scalpel, cut into small pieces, and cultured in DMEM with GIBCO GlutaMAX-I supplemented with penicillin (100 U/mL), streptomycin (100 *μ*g/mL), and amphotericin B (250 ng/mL) (all from Invitrogen/Life Technologies, Carlsbad, CA, USA) at 37°C in humidified 5% carbon dioxide atmosphere for 42 h. Cartilage samples were incubated for 42 h. The cartilage explants were weighted after the incubation and the results were expressed per milligram of cartilage. The culture media were stored at −20°C until analyzed.

### 2.3. Primary Chondrocyte Experiments

Primary chondrocyte experiments were performed as previously described by Koskinen et al. [16]. Briefly, leftover pieces of OA cartilage were processed in the same way as cartilage for tissue cultures (see above). Cartilage pieces were washed with PBS and chondrocytes were isolated by enzymatic digestion for 16 h at 37°C in a shaker by using a collagenase enzyme blend (1 mg/mL Liberase Research Grade medium; Roche, Mannheim, Germany). Isolated chondrocytes were washed and plated on 24-well plates (1.5 × 10^5^ cells/mL) in culture medium (DMEM with supplements; see above) containing 10% fetal bovine serum. Chondrocytes were treated with IL-6 (100 ng/mL) + sIL-6R (100 ng/mL), IL-6 alone, sIL-6R alone, or with IL-17 (50 ng/mL) for 24 h. All obtained from R&D Systems Europe Ltd., Abingdon, UK. The culture media were stored at −20°C until analyzed.

### 2.4. Measurement of YKL-40, IL-6, IL-17, MMP-1, and MMP-3

Concentrations of YKL-40, IL-6, IL-17, MMP-1, and MMP-3 in plasma, SF, and culture media were measured by ELISA with commercial reagents from R&D Systems Europe Ltd., Abingdon, UK (YKL-40, MMP-1 and MMP-3), from Sanquin, Amsterdam, The Netherlands (IL-6) and from eBioScience Inc., San Diego, CA, USA (IL-17). The detection limits were 7.8 pg/mL for YKL-40, 0.3 pg/mL for IL-6, 1.95 pg/mL for IL-17, 19.6 pg/mL for MMP-1, and 7.8 pg/mL for MMP-3.

### 2.5. Statistical Analysis

Results are expressed as mean ± SEM. Differences between groups were tested by unpaired *t*-test or one-way analysis of variance (ANOVA) followed by Dunnett's multiple comparisons test. *P* values less than 0.05 were considered significant. Pearson's *r* was used to analyse correlations. Instat (Graph-Pad Software, La Jolla, CA, USA) and SPSS 21 software (SPSS Inc., Chicago, IL, USA) were used in the statistical analysis.

## 3. Results

### 3.1. YKL-40 Concentrations in Plasma, Synovial Fluid, and Cartilage Culture Medium

**Figure 1:**
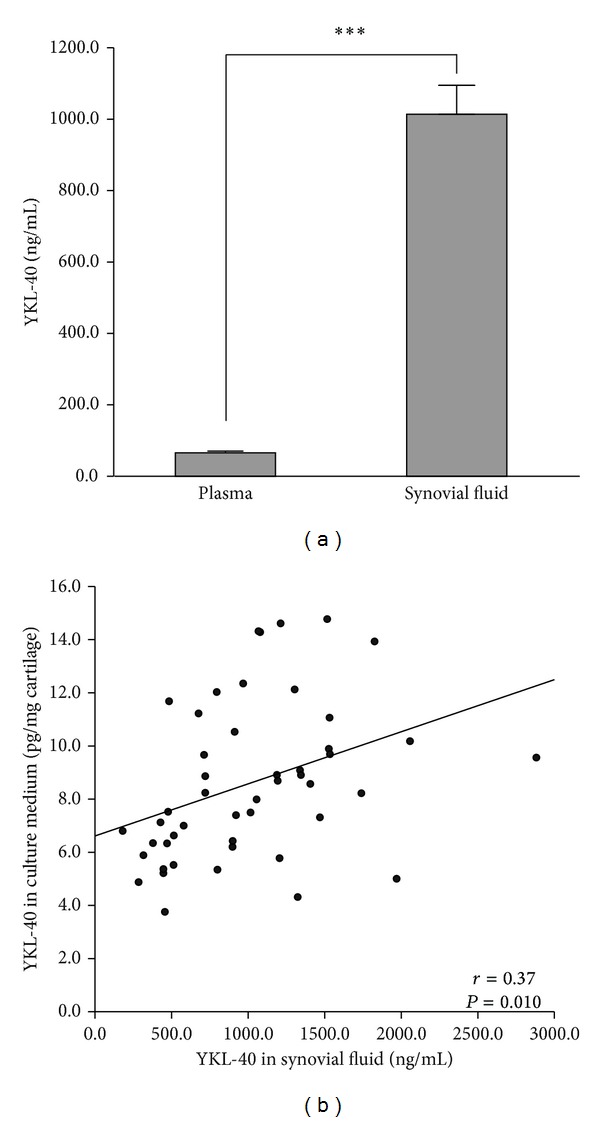
Concentrations of YKL-40 in OA synovial fluid were significantly higher than those in plasma and they correlated with YKL-40 released from cartilage in culture. (a) Mean concentration of YKL-40 in SF was 1027.9 ± 78.3 ng/mL and in plasma 67.2 ± 4.5 ng/mL. Results are presented as mean ± SEM. ***P < 0.001. (b) Scatterplot shows positive correlation between concentrations of YKL-40 in SF and those released into the culture media from OA cartilage during 42 h incubation (*r* = 0.37, *P* = 0.010, *n* = 48).

One hundred OA patients were included in this study (62 females, body mass index (BMI) 30.8 ± 0.6 kg/m^2^, age 70.0 ± 1.0 years; mean ± SEM). SF samples were obtained from 49 patients and the levels of YKL-40 were considerably higher (1027.9 ± 78.3 ng/mL) compared to the plasma levels (67.2 ± 4.5 ng/mL, *P* < 0.001, Figure 1(a)). There were no differences between the genders in plasma or SF levels of YKL-40 (SF: females 1114.1 ± 107.0 ng/mL versus males 879.6 ± 101.6 ng/mL, *P* = 0.151; plasma: females 66.2 ± 5.3 ng/mL versus males 68.9 ± 8.1 ng/mL, *P* = 0.773). YKL-40 concentrations in SF did not correlate with plasma YKL-40, and plasma or SF YKL-40 did not correlate with BMI. Interestingly, SF YKL-40 correlated with YKL-40 released from the cartilage into the culture medium during 42 h incubation (*r* = 0.37, *P* = 0.010, Figure 1(b)), indicating that YKL-40 is produced by OA cartilage.

### 3.2. YKL-40 Correlated with MMP-1 and MMP-3 in Synovial Fluid and in the Cultures of OA Cartilage

**Figure 2:**
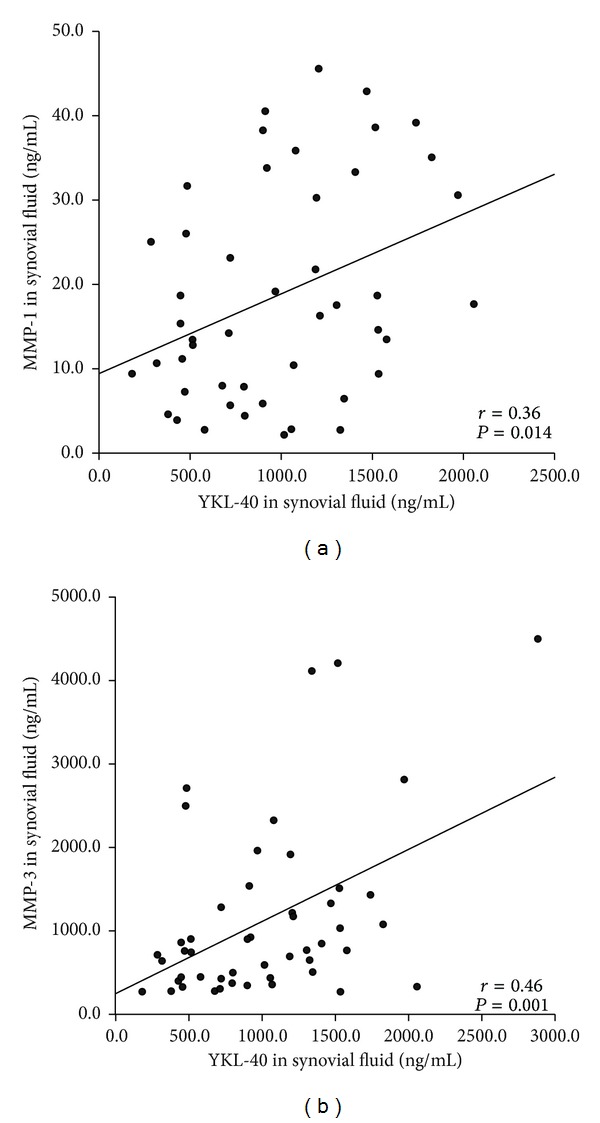
YKL-40 correlated with MMPs in synovial fluid. YKL-40 concentrations correlated positively with those of matrix metalloproteinases (a) MMP-1 and (b) MMP-3 in SF from OA patients (*r* = 0.36, *P* = 0.014 and *r* = 0.46, *P* = 0.001, resp.).

**Figure 3:**
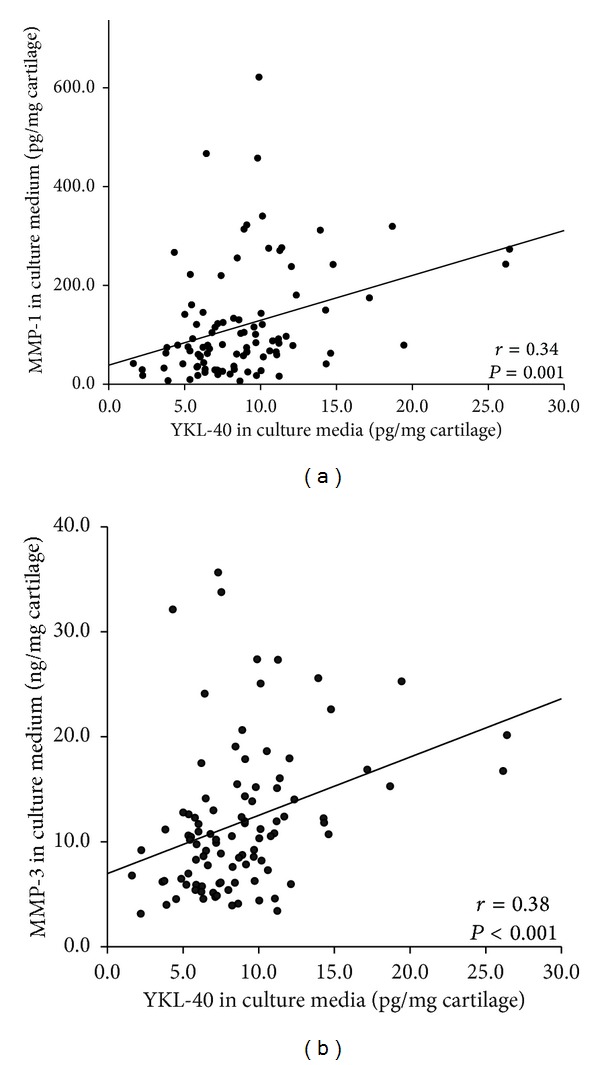
YKL-40 release from cultured OA cartilage correlated with MMP-1 and MMP-3. Cartilage samples from 97 OA patients were incubated for 42 h. Concentrations of YKL-40 released from OA cartilage into the culture media correlated positively to those of (a) MMP-1 and (b) MMP-3 (*r* = 0.34, *P* = 0.001 and *r* = 0.38, *P* < 0.001, resp.).

To assess the role of YKL-40 in OA, concentrations of MMP-1 and MMP-3 were measured in the SF samples and culture media of the cartilage samples, as these enzymes are key mediators of the catabolic events in OA cartilage [17]. Interestingly, in SF YKL-40 levels correlated positively with extracellular matrix degrading MMP-1 (*r* = 0.36, *P* = 0.014, Figure 2(a)) and MMP-3 (*r* = 0.46, *P* = 0.001, Figure 2(b)), but there were no such correlations in the plasma levels suggesting a role for YKL-40 in the intra-articular events in OA pathology. Moreover, YKL-40 released from the cultured OA cartilage correlated positively with MMP-1 (*r* = 0.34, *P* = 0.001, Figure 3(a)) and MMP-3 (*r* = 0.38, *P* < 0.001, Figure 3(b)) release, pointing to the cartilage as the scene for these events.

### 3.3. IL-6 and IL-17 Induced YKL-40 Production in Primary OA Chondrocytes

**Figure 4:**
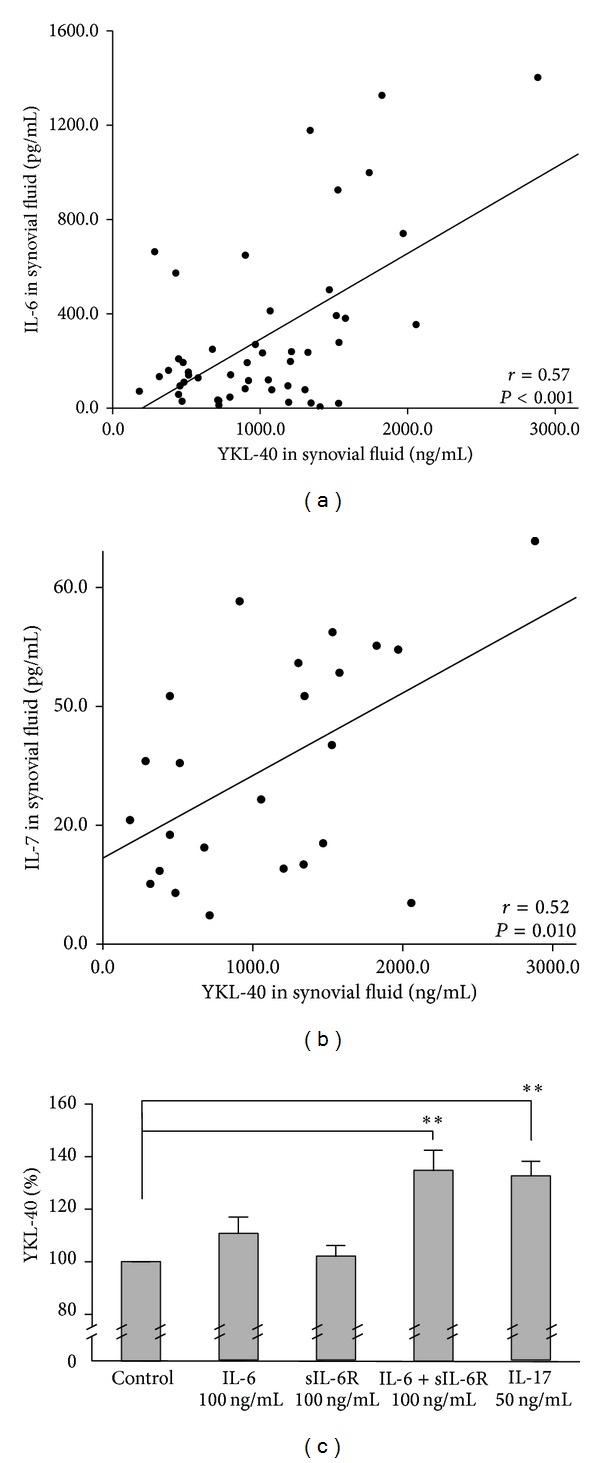
Relation of YKL-40 to proinflammatory cytokines IL-6 and IL-7. In synovial fluid, YKL-40 correlated positively with (a) IL-6 and (b) IL-17 (*r* = 0.57, *P* < 0.001 and *r* = 0.52, *P* = 0.010, resp.). In the case of IL-17, the levels were measured from SF samples from 47 patients but the levels remained below the detection limit in about half of the patients (*n* = 23) and they were excluded from the correlation analysis. (c) In a 24 h incubation IL-6 together with its soluble receptor (sIL-6R) and IL-17 stimulated YKL-40 production by over 30% in primary chondrocytes from OA cartilage. Results are presented as mean ± SEM. **P < 0.01 compared to the control sample.

To investigate the associations of YKL-40 with IL-6 and IL-17 implicated in the pathogenesis of OA, we measured SF levels of these proinflammatory cytokines [12]. Interestingly, SF YKL-40 showed positive correlation with IL-6 (*r* = 0.57, *P* < 0.001, *n* = 49, Figure 4(a)) and IL-17 (*r* = 0.52, *P* = 0.010, *n* = 24, Figure 4(b)). In the case of IL-17, the levels were measured from SF samples from 47 patients but the levels remained below the detection limit in about half of the patients (*n* = 23) and they were excluded from the correlation analysis. To evaluate the possible effect of these cytokines on the production of YKL-40, primary chondrocytes from OA cartilage were incubated with IL-6 + soluble IL-6 receptor (sIL-6R, 100 ng/mL) or with IL-17 (50 ng/mL). Both of these cytokines stimulated YKL-40 production by over 30% during a 24 h incubation, while IL-6 or sIL-6R alone did not influence YKL-40 production (Figure 4(c)).

## 4. Discussion

YKL-40 is abundantly present within the OA joints as reported in the present study. We investigated the role of YKL-40 in OA patients by assessing simultaneously taken blood, SF, and cartilage samples from OA patients. To our knowledge, this is the first study showing that YKL-40 correlates positively with MMP-1 and MMP-3 in the synovial fluid and in cartilage culture medium. Further, YKL-40 correlated with IL-6 and IL-17 in synovial fluid and both IL-6 and IL-17 enhanced YKL-40 production in primary OA chondrocytes.

YKL-40 lacks enzymatic activity and specific receptor is not known, but it has been suggested to be involved in inflammatory processes in arthritis, asthma, COPD, liver fibrosis, and cancer [1]. YKL-40 has been shown to bind to important components in cartilage extracellular matrix, that is, to proteoglycans and collagens, and influence their production and assembly [18]. Further, YKL-40 has been suggested to interact with heparin sulfate side chain of syndecan receptor, a family of cell surface proteoglycan receptors regulating cartilage breakdown and synovial inflammation [19]. These novel findings bring out intriguing prospects. Why YKL-40 levels are elevated within the joint in diseases like OA and RA? Is it just a biomarker reflecting inflammation or is it an active molecule in the pathogenesis of these diseases? Despite the growing interest and studies in recent years, the role of YKL-40 in OA has remained unclear [8].

We report here over 10 fold greater levels of YKL-40 in the synovial fluid than those measured in plasma in OA patients. Further, the YKL-40 level in SF was found to be independent of the level in plasma. In support, 10–15 fold SF levels compared to serum levels have been reported [2, 9] and further, serum YKL-40 concentrations in OA patients were reported to be higher compared to those in healthy persons [2, 9, 10]. However, a study by Rego-Pérez et al. found no difference in serum levels of YKL-40 between OA patients and healthy controls [20]. Within OA joint, YKL-40 is produced into the SF by chondrocytes [2, 6, 7], synovial fibroblasts, and differentiated macrophages [2, 6]. In addition, SF neutrophils [2], osteoblasts, and osteocytes present in osteophytes [7] have been shown to express YKL-40. In the present study OA cartilage explants released YKL-40 into culture medium. Moreover, YKL-40 in the culture medium correlated with YKL-40 in concomitantly obtained SF from the same patient levels suggesting cartilage as a significant source of SF YKL-40 in OA.

In the present study, we show for the first time correlation of YKL-40 with MMP-1 and MMP-3 both in SF and in the culture medium of cartilage explants. Previously, Takahashi et al. have shown correlation of serum YKL-40 to MMP-3 and Volck et al. have shown YKL-40 positive cells within the cartilage to localize in the same zones with MMP-1 and MMP-8 [11, 21]. As OA cartilage produces YKL-40 and the amount of released YKL-40 correlates with MMP-production, it is possible that MMP's release YKL-40 from extracellular matrix of cartilage. In fact, YKL-40 has been suggested to be a secreted protein based on its intracellular localization [2, 21, 22]. Moreover, Volck et al. showed expression of YKL-40 in the pannus-invaded cartilage, but not in the underlying arthritic cartilage [2]. Possible reasons not to detect YKL-40 in the extracellular matrix are low concentrations of YKL-40 within cartilage extracellular matrix and/or prevention of detection due to YKL-40 binding to extracellular matrix compounds [2, 6, 22]. Interestingly, despite the fact that YKL-40 lacks enzymatic activity of true chitinases, the hydrophobic binding cleft is preserved and YKL-40 has been shown to bind not only to chitin, which is a polymer of N-acetylglucosamine (a common structural molecule in fungi, invertebrates and in extracellular matrix proteoglycans in human cartilage), but also to heparin and noncarbohydrate collagens I, II, and III, the major constituents of extracellular matrix present in for example, cartilage [23–25].

Another explanation to the correlation of YKL-40 and MMPs in the culture medium of OA cartilage explants is that their production is stimulated by a common factor. A clue to this direction was our finding showing correlation of SF YKL-40 levels with proinflammatory IL-6 and IL-17 levels as supported by Johansen et al. in respect to IL-6 [9]. Causality was confirmed in our study, as these cytokines enhanced YKL-40 production by over 30% in primary chondrocytes from OA cartilage. These findings suggest that in OA YKL-40 is not released from the cartilage matrix by MMPs but rather produced by chondrocytes activated by inflammatory stimulus.

Strength of the present study is the availability of simultaneous samples of blood, SF, and cartilage from OA patients as the current knowledge is that OA related changes in the joint tissues are reflected to the synovial fluid and that chondrocytes take active part in the cartilage degradation in OA. Limitation of the study is the lack of healthy controls and that the patients recruited had later stage OA as they underwent a total knee replacement surgery. A correlation between serum or SF YKL-40 and OA grade has not been found in earlier studies [6, 26]. Connor et al. and Johansen et al. have shown that the level of YKL-40 expression in the cartilage is related to OA grade, but there are, however, also controversial results [7, 22, 27, 28].

## 5. Conclusions

The present study shows that in OA patients YKL-40 is produced by articular chondrocytes, and that its production is increased by inflammatory cytokines IL-6 and IL-17. Moreover, the levels of intra-articular YKL-40 correlate with cartilage matric degrading enzymes MMP-1 and MMP-3. The results introduce YKL-40 as a factor associated with inflammatory and catabolic processes in OA joints and encourage further studies on the role of YKL-40 in the pathogenesis of OA.
